# Comparison between complete genomes of an isolate of *Pseudomonas syringae* pv. actinidiae from Japan and a New Zealand isolate of the pandemic lineage

**DOI:** 10.1038/s41598-018-29261-5

**Published:** 2018-07-19

**Authors:** Russell T. M. Poulter, Joycelyn Ho, Thomas Handley, George Taiaroa, Margi I. Butler

**Affiliations:** Department of Biochemistry, University of Otago, Dunedin, New Zealand

## Abstract

The modern pandemic of the bacterial kiwifruit pathogen *Pseudomonas syringae* pv actinidiae (Psa) is caused by a particular Psa lineage. To better understand the genetic basis of the virulence of this lineage, we compare the completely assembled genome of a pandemic New Zealand strain with that of the Psa type strain first isolated in Japan in 1983. Aligning the two genomes shows numerous translocations, constrained so as to retain the appropriate orientation of the Architecture Imparting Sequences (AIMs). There are several large horizontally acquired regions, some of which include Type I, Type II or Type III restriction systems. The activity of these systems is reflected in the methylation patterns of the two strains. The pandemic strain carries an Integrative Conjugative Element (ICE) located at a *tRNA-Lys* site. Two other complex elements are also present at *tRNA-Lys* sites in the genome. These elements are derived from ICE but have now acquired some alternative secretion function. There are numerous types of mobile element in the two genomes. Analysis of these elements reveals no evidence of recombination between the two Psa lineages.

## Introduction

*Pseudomonas syringae* pv. actinidiae (Psa) is an emerging bacterial plant pathogen, the causal agent of bacterial canker of kiwifruit. The pathovar includes distinct lineages of varying virulence and global distribution, described as biovars^1,2^. Strains associated with the first disease observation in Japan (1984–1989) and in Italy (1992) are almost genetically identical and have been assigned to biovar 1 (Psa1)^3^. The strains responsible for the current pandemic in Europe, New Zealand and Chile belong to biovar 3 (Psa3)^4^. The ongoing evolution of *P*. *syringae* pv. actinidiae has been shown to be driven by horizontal gene transfer, as well as defined chromosomal changes, partly in response to anthropogenic selective pressures^5–8^. A genetic comparison between the Psa1 and Psa3 lineages will contribute to understanding the drivers of virulence in this economically important pathogen, as well as further characterize patterns of evolutionary diversification in Psa.

Here we describe a comparison between the complete genome of the earliest Psa lineage identified, Psa1 from Japan in 1984^9^, and that of a strain from the recent global pandemic lineage Psa3, isolated New Zealand in 2010^10^. These genomes were assembled from PacBio or SMRT (single molecule real time) long read sequences, and error corrected using Illumina short reads.

## Results

### Comparison of complete genomes

The genomes of two Psa strains, ICMP9853 and ICMP18708, were sequenced and assembled. ICMP9853, also known as Kw1, was isolated in Japan at the same time and from the same prefecture as the type strain ICMP9617/KW11^9^. ICMP18708 was isolated in New Zealand in 2010^10^, near the time of the Psa incursion into New Zealand and belongs to the pandemic lineage. Comparison of the assembled genomes of ICMP9853 (Psa1), and ICMP18708 (Psa3), and sequences from related pseudomonad strains reveals multiple re-arrangements, horizontal acquisition of sequences from other pseudomonads, and the active movement of a variety of mobile elements.

The genome of *P*. *syringae* pv. actinidiae Psa1, ICMP9853 is composed of a circular chromosome 6,439,609 bp in size and two small plasmids, p9853-A (34,963 bp) and p9853-B (32,947 bp) (Genbank accessions CP018202, CP018203 and CP018204, respectively). In comparison, the chromosome of Psa3 strain ICMP18708 is slightly larger at 6,555,571 bp, and is accompanied by a single 74,432 bp plasmid, p18708 (CP012179 and CP012180).

**Figure 1:**
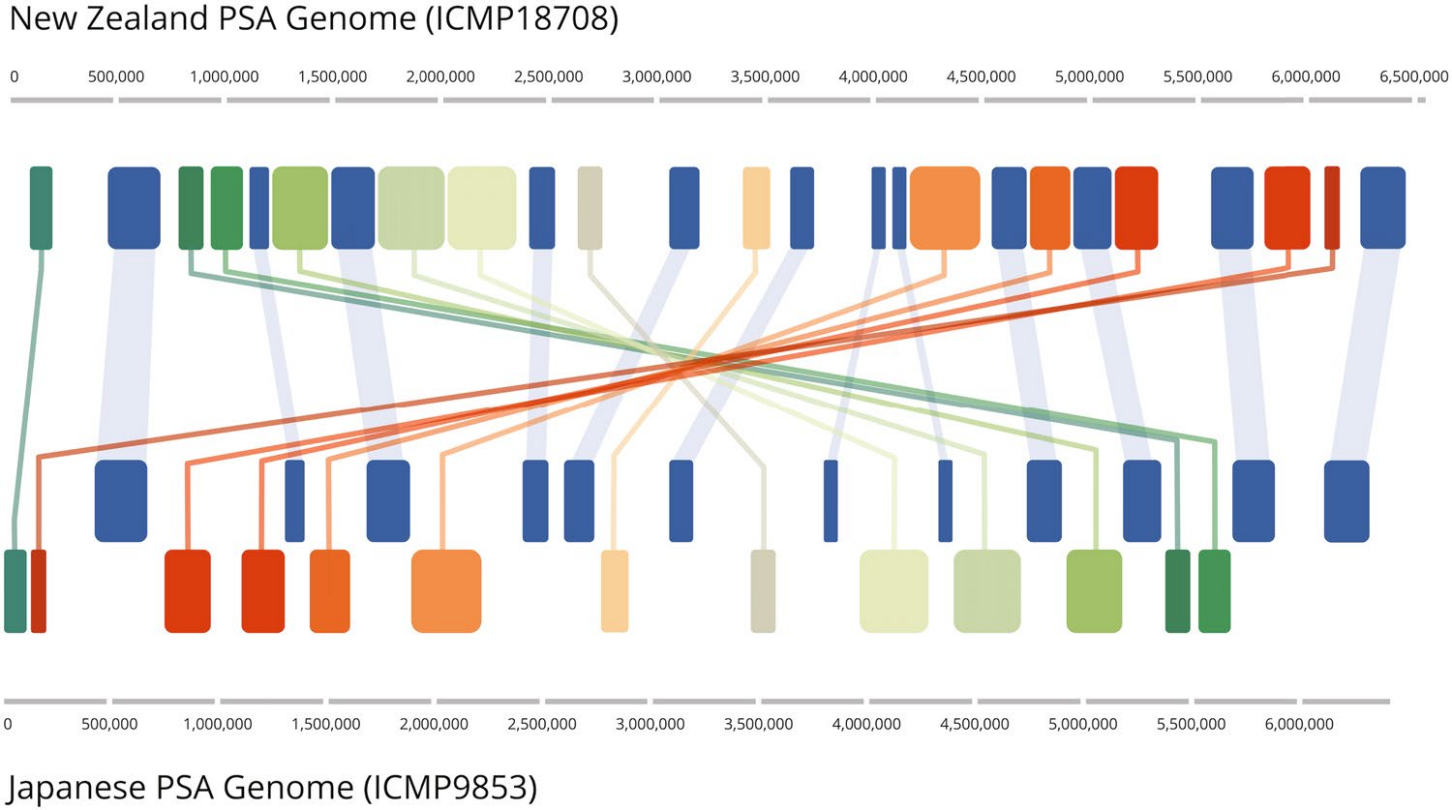
ProgressiveMauve alignment of the ICMP18708 (Psa3) genome with the ICMP9853 (Psa1) genome. Coloured blocks each represent a locally co-linear block containing no apparent re-arrangements. Blocks below the centre line indicate regions that align in the reverse complement (inverse) orientation.

**Figure 2:**
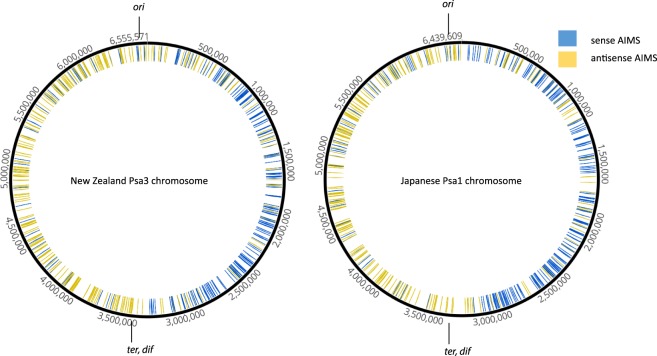
The occurrence of an eight-base pair AIM sequence (5′ GAGCAGGG), on the sense and anti-sense strands of the New Zealand (Psa3) and Japanese (Psa1) genomes.

Comparison of the complete Psa1 and Psa3 genomes using progressiveMauve Indicates that there have been multiple rearrangements in the two strains (Fig. 1). The Mauve alignment is a linear representation of the circular genomes. The first gene in the alignment is *dnaA* (the origin of replication protein); the last gene is the *rpmH* (50 S ribosomal protein L34). The linear representation splits the *oriC* region which contains four perfect matches to the TTATCCACA DnaA binding sites described in the *oriC* of *Pseudomonas putida* and *P*. *aeruginosa*^11^ and *Pseudomonas* UW4^12^. The genome rearrangements retain the origin (*oriC*) and terminus of replication (*ter*) at opposite, polar, positions in the circular molecule. The translocation events are probably constrained by the FtsK/AIMS (architecture imparting sequences) system. The FtsK protein recognises specific DNA motifs termed AIMS or FtsK-orientating polar sequences (KOPS)^13,14^. AIM sequences are orientated towards the *dif* site, which is located in the *ter* region. The motifs act as signposts that direct FtsK to the *dif* site where it is required for chromosome dimer resolution prior to cell division. Genome rearrangements have been hypothesized to be limited to those which have an AIMS compatible orientation. Multiple AIM sequences have been described from *Pseudomonas aeruginosa*^14^. Figure 2 shows the occurrence of one example, the *P*. *aeruginosa* eight base pair AIM sequence 5′ GAGCAGGG, on the sense and anti-sense strands of the New Zealand (Psa3) and Japanese (Psa1) genomes. The other proposed *P*. *aeruginosa* AIMS show the same clear pattern in both Psa genomes (Supplementary Figure 1). Supplementary Figure 1 summarises the distribution of all identified eight *P*. *aeruginosa*/Psa AIMS, with each following the observed skew. It has been suggested that the frequency of AIMS increases towards the *ter*^14^; the Psa genomes show some increase (Fig. 2). In summary, the numerous rearrangements or translocations present in these genomes have not disturbed the orientation of the AIMS or altered the polar positions of the *oriC* and *ter*.

### Plasmids as a means of horizontal gene transfer

The Japanese Psa strain ICMP9853 maintains two plasmids, a 34,963 bp plasmid p9853A (GenBank CP018203) and a 32,947 bp plasmid p9853B (GenBank CP018204) (Supplementary Figure 2). The New Zealand strain, ICMP18708, has a single plasmid, p18708, of 74432 bp (Supplementary Figure 3). All three plasmids carry multiple internally mobile elements such as Insertion Sequences (IS). The plasmids p9853A, p9853B and p18708 are possibly mobilisable since they carry a relaxase gene, required for nicking the double stranded plasmid DNA prior to conjugative transfer. However, the plasmids are not self-transmissible. Neither of the plasmids from the Japanese strain encodes any conjugal transfer proteins. The p18708 plasmid from the New Zealand strain encodes five conjugal transfer proteins, however there are no genes likely to encode a surface-exposed sex pilus.

The Psa1 plasmid p9853A carries a subset of genes with homology to genes carried by the Psa3 plasmid p18708. Both plasmids carry, in the same order, genes showing sequence similarity to *parA* (>99% identity at the protein level), toxin/antitoxin, *mobC*, *mobB*, *mobA/relaxase* (95% identity at the protein level) and *repA* (95% identity at the protein level). Excluding the mobile elements, none of the genes on p9853B show any significant similarity to p9853A. Only one gene on p9853B, encoding a putatively functional type III effector, HopAV1, shows any sequence similarity to p18708. The *hopAV1* sequence on p9853B is flanked by sequences from a Tn3 type transposon and it is probable that this sequence is a recent addition to the Psa1 plasmid. This suggests the *hopAV1* gene may convey a selective advantage for Psa1. In contrast, on p18708, the *hopAV1* is interrupted by the insertion of a miniature inverted-repeat transposable element (MITE) of 102 bp and is likely non-functional. The HopAV1 protein is therefore apparently not a part of the virulence mechanism of Psa3. In Europe, it has been shown that transposition resulting in ISPsy insertions has inactivated genes in the *hrp* cluster of two field isolates of Psa3^8^. These transposition derived *hrp* mutations also alter the virulence of Psa3.

The plasmids are likely to modify the virulence of the Psa. The Japanese (Psa1) plasmid p9853B encodes various type III effector genes (*hop AV1*, *hopPtoH*, *hopX*), and p9853A encodes diguanylate cyclase (involved in biofilm formation and persistence) and a methyl accepting chemotaxis gene. Methyl accepting chemotaxis proteins are used by pseudomonads to orientate themselves in the phyllosphere. The New Zealand Psa3 plasmid, p18708, also carries a number of genes likely to affect the phenotype; for example, a distinct methyl-accepting chemotaxis protein, the LuxR quorum sensing protein and several biosynthetic genes (including glutamine amidotransferase and both subunits of anthranilate synthase).

A previous analysis using short reads^15^, described the Japanese strain ICMP9617 as having a single plasmid of 30,848 bp (GenBank CM002754). This proposed 30,848 bp plasmid corresponds approximately to plasmid p9853B (32,947 bp). Our SMRT sequencing indicates that the CM002754 assembly is missing an ISPsy31 and an ISPsy39 element. The previous analysis also incorrectly incorporated the sequence corresponding to p9853A into the main chromosome of ICMP9617 (GenBank CM002753) at position 6289347–6323951. PCR analyses indicate that there is no plasmid (or other sequence) incorporated at this position in ICMP9853 or ICMP9617.

### Horizontal transfer and Restriction-Modification systems

The comparison of the New Zealand Psa3 (ICMP18708) and the Japanese Psa1 (ICMP9853) genomes revealed numerous horizontal gene transfer events. Horizontal gene transfer is an important part of evolution, providing the recipient cell with novel genes that can contribute to new phenotypes and potentially improved fitness. Interestingly, the horizontal transfers include the acquisition of multiple restriction modification (R-M) systems. Restriction modification systems are a defence mechanism against extrachromosomal DNA and elements such as phage, recognising and cleaving specific DNA sequences. R-M systems are also implicated in global changes in methylation patterns and virulence^16,17^.

There is evidence that suggests that some Psa restriction modification systems have been selectively disrupted by mobile elements (Table 1 and Supplementary Figure 4).

**Table 1:** Restriction-Modification systems and dam/dcm methylases present in the genomes of Psa3 (ICMP18708) and Psa1 (ICMP9853).

REBase name	type	Psa3, ICMP18708	position in genome	Psa3, Hym1	Psa3, Jilo4
M.Psy708NZORF25P	Type I	AGCANNNNNGTC	6,593–12,826	present	absent
M.Psy708NZORF130P	Type I	disrupted by ISPsy34	33,116–41,522	present but disrupted	present, no disruption
M.Psy708NZORF855P	Type II	CTCGAG	181,850–202,419		
RM.Psy708NZORF5205P	Type II	non-functional homologue of RM-Psy9853ORF25530P, disrupted by two MITEs and ISPsy28	1,105,031–1,107,495		disrupted by one MITE and ISPsy28
M.Psy708NZORF8545P	Type III	disrupted by in-frame stop and a MITE	1,837,995–1,845,322		disrupted by in-frame stop
M.Psy708NZORF24760P	dcm	CC(A/T)GG	5,495,131–5,496,546		
		Sequence similarity suggests this a dcm methylase; it is present within the Pac_ICE1 element			
**REBase name**	**type**	**Psa1, ICMP9853**	**position in genome**		
M.Psy9853I/Psy9853IP	Type II	CTGCAG	1,702,030–1,704,452		
RM.Psy9853ORF25530P	Type II	Deletion of 951 bp, homologue of RM-Psy708NZORF5205P	5,320,330 - 5,318,258		
M.Psy9853II/Psy9853IIP	Type III	TCCACC	6,024,961–6,029,546		
M.Psy9853ORF28855P	dam	GATC	6,032,376–6,033,170		
		Sequence similarity suggests this is a dam methylase; it is present in an integrated prophage			

### New Zealand Psa3 R-M systems

The New Zealand strain contains two putatively active R-M systems (one Type I and one Type II). In addition, the New Zealand Psa3 contains three non-functional restriction modification systems (two Type II and a Type III) disrupted by mobile elements. The comparison of the New Zealand Psa3 with the Japanese Psa1 highlighted a region of approximately 45 kb long (positions 6,345 bp–51,914 bp) present in the New Zealand strain but completely absent from the Japanese strain. The boundaries of the suggested horizontal transfer were defined by the areas of sequence conservation that flank the acquisition. The region contains two putative Type I restriction modification systems (ORF25 at positions 6,593–12,826 and ORF130 at position 33,116–41,522). The methylation profile gathered from SMRT sequencing data analysis indicates only one Type I restriction modification motif (AGCANNNNNGTC) is methylated, indicating only one of the two annotated restriction modification systems is functional. Further to this, ORF130 has an IS element (ISPsy34) interrupting the gene encoding the methyltransferase subunit, making the operon defective.

**Figure 3:**
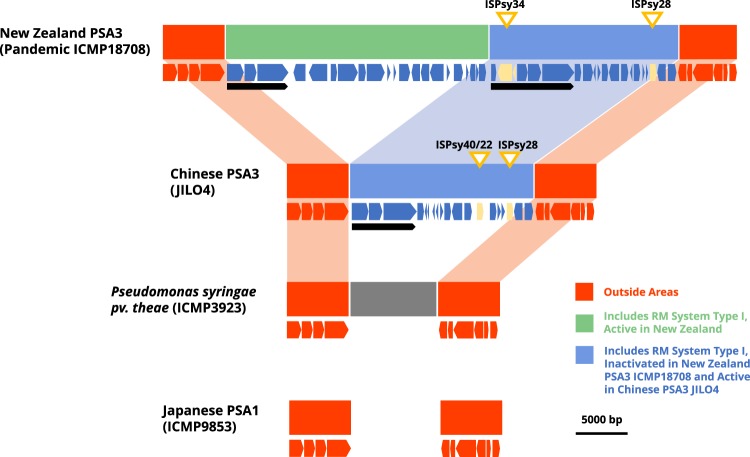
The acquisition of Type I restriction modification systems in Psa3. Representation of the first 58,000 bp of the New Zealand Psa3 genome (ICMP18708) compared to the homologous regions in a Chinese ccB isolate of Psa3 (Jilo4), in *P*. *syringae* pv. theae (ICMP3239) and in Psa1 (ICMP9853). The red boxes correspond to regions present in all four strains, the dark blue boxes represent one horizontally acquired region that includes a Type I restriction system (black arrow) which is shared by both Psa3. The second horizontally acquired region (a green box in ICMP18708) also includes a Type I restriction system (black arrow). Data from Jilo4 and *P*. *syringae* pv. theae are contained in draft assemblies of 200 contigs longer than 5,000 bp.

In an effort to determine whether these R-M systems represent gene gain events acquired by Psa3 and not deleted from Psa1, we examined the genomes of other related but non-pandemic Chinese isolates of Psa3, as well as the genome of *P*. *syringae* pv. theae (ICMP3923), the most closely related *P*. *syringae* pathovar to Psa. Isolate Hym1, a member of clonal complex A (ccA) of Psa3^18^ contains the same arrangement as the pandemic New Zealand Psa3 isolate, including both the functional (ORF25, AGCANNNNNGTC) Type I restriction system and the disrupted ORF130 Type I restriction system. In contrast, isolate Jilo4 of Psa3 (ccB)^18^ lacks completely the (AGCANNNNNGTC) Type I restriction system and has an apparently functional (undisrupted) version of the ORF130 Type I restriction system (Fig. 3). *P*. *syringae* pv. theae carries neither of the Type I restriction systems in this region (Fig. 3). These data all suggest that the R-M systems were gene gain events in Psa3.

This suggests that there have been two, separate, horizontal transfers of Type I restriction systems into this region of the Psa3 genome. The first acquisition is present in all Chinese Psa3 but has become inactivated in Hym1 (a member of the ccA group of Chinese Psa3; 18) and in the pandemic strains. The second acquisition is present only in Hym1 and the pandemic Psa3. This second acquisition is probably plasmid derived. There are several plasmid-associated genes embedded in the region, such as chromosome partitioning proteins and death-on-curing proteins.

The source of both acquisitions is likely to be the species *Pseudomonas amygdali* pv. lachrymans. A protein BLAST search of both Type I restriction modification gene sets revealed 100% query coverage and 99% identity to sequences encoded in *P*. *amygdali* pv. lachrymans.

In addition to containing an active Type I restriction modification system, the New Zealand strain also contains an apparently active Type II restriction modification system. The methylation profile gathered from SMRT sequencing data indicates the restriction recognition motif is CTCGAG. This restriction enzyme is present in a 20 kb region (positions 181,850–202,419) in the New Zealand strain but absent from ICMP9853. A protein BLAST search with the amino acid sequence of the methyltransferase gene showed a 100% coverage and identity of 99% to *Pseudomonas syringae* pv. theae. A second Type II restriction modification system, RM.Psy708NZORF5205P, is disrupted by three mobile element insertions and is unlikely to be active (Table 1). There is a homologue, also inactive, of this gene in the Japanese Psa1 genome.

The New Zealand strain also contains a disrupted Type III restriction enzyme (position 1,838,271 bp to 1,844,628 bp) in a putative horizontally transferred region. This region is absent from the Japanese strain. The horizontally acquired region is 7 kb long and includes a methyltransferase disrupted by an in-frame stop and a miniature inverted-repeat transposable element (MITE) together with a Type III restriction endonuclease (position 1,837,995 to 1,845,322). This Type III restriction modification system, including the in-frame stop and MITE, is also present in the Chinese Psa3 strain Hym1. In contrast, the Chinese Psa3, Jilo 4, contains the in-frame stop but not the MITE (Table 1). The most closely similar sequence to that of the Type III restriction endonuclease comes from *Pseudomonas stutzeri* with 99% query coverage and sequence 97% identity.

### Japanese Psa1 R-M systems

The Japanese strain contains two active R-M systems (one Type II and one Type III), neither of which is present in the New Zealand Psa3 (Table 1). A Type II restriction modification system recognising CTGCAG sites is found between 1,702,030 and 1,704,452 in a 29 kb genomic island. Genes in this island include a chromosome partitioning protein and P-type conjugative transfer protein, suggesting this is a remnant of a conjugative element such as a plasmid or an integrative and conjugative element (ICE). The sequence is absent from the New Zealand strain; this allows the borders of the element to be defined as between positions 1,687,590 and 1,717,087 in the Psa1 Japanese genome. A protein BLAST search of the methyltransferase amino acid sequence revealed its closest match is to *Pseudomonas syringae* pv. maculicola, with a query cover of 100% and sequence identity of 99%.

A Type III restriction modification system, recognising TCCACC sites, is found in an approximately 50 kb region between positions 6,021,188 and 6,073,136 in the Psa1 genome. The restriction modification genes are located between positions 6,024,961 and 6,029,546. A number of phage associated genes are found within the 50 kb region indicating that the restriction modification system was likely introduced via transduction. The methyltransferase protein sequence has a perfect match to sequences in *P*. *syringae* pv. savastanoi and *P*. *syringae* pv. maculicola. Its cognate restriction subunit sequence matched most closely to sequences in *P*. *syringae* pv. savastanoi with a query cover of 100% and 99% sequence identity.

The Psa1 genome contains a homologue of the Type II RM-Psy708NZORF5205P found in ICMP18708. This gene (RM.Psy9853ORF25530P) in ICMP9853 is severely truncated and unlikely to be functional.

### Occurrence of dam- and dcm methylases

In addition to the methyltransferases associated with R-M systems, there are methyltransferase genes in both Psa1 and Psa3 genomes that are not accompanied by a cognate restriction enzyme. Such so-called ‘orphan’ methyltransferases are known to regulate diverse host cell processes^19,20^. There is a *dcm* methylase in Psa3 (absent in Psa1) carried on the integrative, conjugative element or ICE (Pac_ICE1). The bacterial *dcm* methylases recognise CC(A/T)GG sequences and have been implicated in gene regulation^21^. In contrast, in the Psa1 genome, there is a *dam* methylase carried on a prophage which is absent in Psa3. Dam methylase, which recognises the sequence GATC, is critical in mismatch repair of replicated DNA. In addition, dam methylases have been implicated in bacterial virulence, for example in *Salmonella enterica*, dam methylase activates invasion genes in the pathogenicity island SPI-1 while the fimbrial operon *std* was repressed by dam methylation^22^.

### Evolution of ICE and exapt_ICE

Integrative, conjugative elements (ICEs) are a diverse group of mobile elements found in both Gram-positive and Gram-negative bacteria. ICEs are self-transmissible elements that encode a full complement of machinery necessary for conjugation^23^. ICEs can promote their own mobilisation and potentially that of ‘hitch-hiking’ genetic elements, and thus contribute to the horizontal transfer of virulence determinants, antibiotic resistance genes and other bacterial traits^24^. The sequence of different strains of the pandemic Psa3 are very similar except for the diverse ICEs that they carry. The New Zealand strain ICMP18708 carries Pac_ICE1, recent Psa3 isolates from Europe carry Pac_ICE2 and Psa3 isolates from Chile carry Pac_ICE3^25^. These ICEs are integrated at one of two *tRNA-Lys* target sites. New Zealand isolates carry the Pac_ICE1 adjacent to the *tRNA-Lys* at position ~5.5 Mb and have an unoccupied *tRNA-Lys* at position ~1.73 Mb. In contrast, the European isolates have Pac_ICE2 integrated at the target *tRNA-Lys* at the 1.73 Mb site while the other (~5.5 Mb) target *tRNA-Lys* is unoccupied^8,25^.

**Figure 4:**
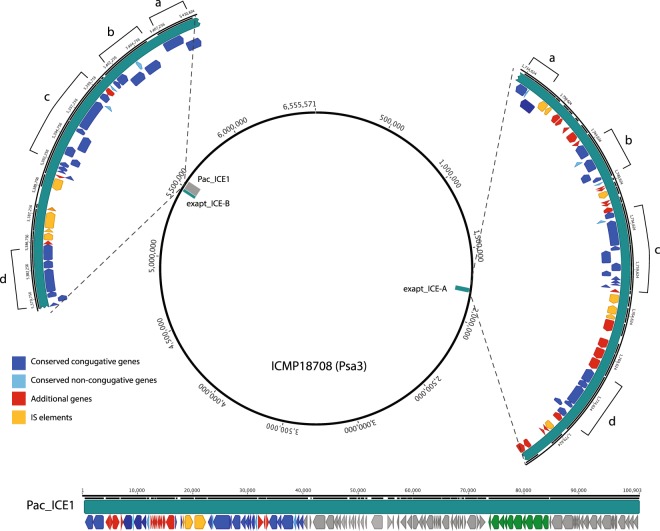
Exapt_ICEs in the New Zealand Psa3 genome. A description of the position in the ICMP18708 genome, and the gene content of exapt_ICE-A and exapt_ICE-B. The arrows represent open reading frames, which are coloured according to their predicted protein products. Green arrows in Pac_ICE1 represent the *pil* gene array, grey arrows represent ORFs not present in the exapt_ICEs. Sections marked with the same letter correspond to homologous sequences.

Comparison of New Zealand and European Psa enables the exact boundaries of the integrated ICE to be defined by comparison of the occupied and unoccupied sites. Following integration, Pac_ICE1 is flanked by a pair of *tRNA-Lys*, one at 5,511,679–5,511,754 and another *tRNA-Lys* at 5,410,906–5,410,827, representing the *att*L and *att*R sites. The first gene in Pac_ICE1 encodes a XerC tyrosine recombinase involved in site specific excision/integration. The second gene encodes a TraI relaxase involved in nicking the double stranded circular ICE prior to conjugative transfer. Pac_ICE1 carries a full complement of genes required for conjugation, including both *tra* and *pil* genes (Fig. 4). There are, in ICMP18708 (and all the pandemic Psa3), immediately adjacent to both the *tRNA-Lys* sites (~1.73 Mb and ~5.54 Mb), additional *xerC* and *traI* related sequences together with other genes suggesting the presence of remnants of ICE elements. These remnant ICE will be referred to as exapt_ICE-A and exapt_ICE-B. These remnant ICE sequences are identical in both European and New Zealand Psa3. The Japanese strain, ICMP9853, has two simple *tRNA-Lys* genes without any ICE or exapt_ICE remnants. It is possible, using the Japanese sequence, to determine the exact limits of the exapt_ICE sequences. The first gene encoded by exapt_ICE-A (adjacent to the *tRNA-Lys* at ~1.73 Mb) is a full length *xerC* tyrosine recombinase followed by a full length *traI*. The first gene encoded by the exapt_ICE-B (adjacent to the *tRNA-Lys* at ~5.54 Mb) is a truncated *xerC* tyrosine recombinase followed by a truncated *traI*. The remnant exapt_ICEs do not end in any *tRNA-Lys* related sequence. To clarify this situation a comparison of Pac_ICE1 and the two remnant exapt_ICEs was performed.

The exapt_ICE-B is the smaller remnant; it is 31 kb in length(5,410,875–5,378,923) (Fig. 4). Exapt_ICE-B includes an ISPsy34 and two ISPsy32 insertion sequences. The remaining sequence is devoted almost entirely to genes involved in conjugal pore formation or related conjugative functions. This includes not only genes such as *traG*, but also a lytic transglycosylase required to allow the conjugal pore to form, as well as thioredoxin and protein disulphide isomerase which are needed to allow correct folding of proteins as they pass through the pore. Exapt_ICE-B is clearly not self-transmissible since it lacks the pilus genes found on Pac_ICE1. It is also difficult to see how it could be transmissible using the functions of other ICEs since there is only a *tRNA-Lys* adjacent to the *xerC* and no *tRNA-Lys* attachment (*att*) site at the other end of the element. This exapt_ICE, therefore, cannot be excised from the genome. It is compelling that all the genes annotated as relating to conjugal pore formation in Pac_ICE1 are retained in the exapt_ICE-B (their order is also conserved).

Exapt_ICE-A extends from 1,733,936 to 1,784,329 (~50 kb). As in exapt_ICE-B, there is no evidence of a second flanking *tRNA-Lys* attachment (*att*) site that would be required for excision or evidence of pilus genes. Exapt_ICE-A includes two ISPSy31 and three ISPsy32 insertion sequences. It includes all the conjugative genes found in exapt_ICE-B and Pac_ICE1. These genes occur in the same order in all three elements. The DNA sequence of the conjugative genes suggests a close similarity between the exapt_ICE-A and exapt_ICE-B elements. For example, the *traG* genes of exapt_ICE-A and exapt_ICE-B encode proteins with >99% identity (two amino acid differences in the 719 amino acid protein). They are less similar to the Pac_ICE1 *traG* (97% at the protein level). In addition to conjugative genes (and mobile elements), exapt_ICE-A has a number of ‘cargo’ genes including those encoding an UmuC DNA polymerase, a carbon storage regulator, amidinotransferase, serine kinase and several type III effectors. In summary, the exapt_ICE elements appear to be non-mobilisable elements that retain a shared, complete complex set of genes required for conjugal pore formation but few additional functions. The possible role of the exapt_ICEs will be considered later, but one interesting possibility is that the exapt_ICE system produces membrane pores to allow the secretion of DNA or of effector proteins involved in pathogenesis^26,27^.

The absence of ICE and exapt_ICEs in Psa1, and the presence of these elements in Psa3 may influence the virulence of these biovars. For example, Pac_ICE1 encodes several methyl-accepting chemotaxis proteins and an alginate biosynthesis transcriptional activator. The Pac_ICE1 also encodes UmuD, UmuC and a single stranded DNA binding protein. It is believed that replication by Pol V (also known as UmuC), in the presence of UmuD, RecA, and ssDNA-binding protein, is the basis of highly mutagenic chromosomal SOS untargeted mutagenesis^28^.

### Further horizontal exchange

In addition to the examples of horizontal transfer that resulted in the acquisition of new sequences, there are numerous examples of the acquisition of homologous sequences via horizontal transmission. For example (Supplementary Figure 5) shows the comparison of the ICMP18708 and ICMP9853 genomes between positions 89877 and 98609 of the New Zealand Psa3 genome. Whereas eight of the ORFs in this region show few or no SNPs, the 1898bp cystathionine gamma synthase gene (93,414–95,312) shows 68 SNPs. This abundance of SNPs is not due to intense selection pressure, because 55 of the SNPs are neutral codon changes. It seems likely therefore, that this sequence is due to the horizontal acquisition and incorporation by homologous recombination of a sequence from a different bacterium.

One, possibly intact, prophage was identified in the genome of ICMP18708 (Psa3) at positions 290,350 to 323,480. A prophage is also present in the genome of ICMP9853 (Psa1) at positions 6,021,806 to 6,074,983; however, it is disrupted with multiple mobile elements. Island Viewer predicted multiple genomic islands in both Psa genomes; many represent regions in which a number of insertion sequences or transposons have accumulated.

### Mobile elements

The repetitious mobile elements of bacterial genomes are important drivers of evolution, as demonstrated in the analyses of the R-M systems of Psa. Mobile DNA elements are abundant and varied in the genomes of both Psa1 and Psa3. This is illustrated in the results of a blastn search of the first 1 Mb of the ICMP18708, Psa3, genome compared to the complete chromosome (Supplementary Figure 6). In addition to their biological significance, mobile, repetitious elements cause serious problems with genome assembly. For these reasons, it is important to have an accurate description of the mobile DNA repertoire of a bacterial genome.

The most numerous repeated element is a 102 bp MITE (miniature inverted terminal repeat element), present at 72 loci in the core genome of Psa3 and 6 loci on plasmid p18708. The insertion of this MITE has inactivated the *hopAV1* gene in the Psa3 plasmid, a Type II RM gene and a Type III methylase gene. This indicates that this MITE is being transposed, probably by the activity of an ISPsy25. There is no MITE apparent in the genome of Psa1.

Representatives of 12 families of insertion sequences (IS) can be found in the genomes of both ICMP18708 (Psa3) and ICMP9853 (Psa1). There are multiple, distinct IS from single families: for example, the IS3 family has three complete elements (ISPsy28, ISPsy31 and ISPsy37) which are each present in both Psa3 and Psa1 (Table 2). The most abundant IS are ISPsy32 (IS630 family) with 60 copies in ICMP18708, and ISPsy31 (IS3 family) with 88 copies in ICMP9853. There are instances where a particular IS is abundant in one genome but absent from the other. For example, there are 31 copies of ISPsy38 in ICMP9853, but none in ICMP18708. There are no ISPsy17 elements in Psa1 (ICMP9853) but 11 copies in ICMP18708.

**Table 2:** Examples of mobile DNA elements in Psa3 (ICMP18708) and Psa1 (ICMP9853).

name	size (bp)	copy no. Psa3	copy no. Psa1	Element type or IS family	closest example in GenBank (DNA match)
Tn6212	16316	1	0	XerC	*Ps* pv. syringae, (99%), *Ps* pv. tomato (99%)
ISPsy35	1701	3	1	IS110	*Ps* pv. theae (99%), *Ps* pv. actinidifoliorum (99%), *Ps* pv. viburni (99%)
ISPpu10-like	nd	0	1	IS110	
ISPsy36	1658	21	40	IS1182	*P*. *avellanae*, *Ps* pv. theae, Psa5 (all 100%)
ISPsy40	2644	14	7	IS21	*Ps* pv. actinidifoliorum (99%)
ISPsy41	2575	3	26	IS21	*P*. *mandelii* (91%), *P*. *veronii* (91%)
ISPsy20	2572	0	1	IS21	
ISPsy17	1374	11	0	IS256	*Ps* pv. maculicola (96% id)
ISPsy31	1667	54	88	IS3	*P*. *avellanae*, *Ps* pv. theae (100%)
ISPsy28	1292	19	11	IS3	*P*. *avellanae*, *Ps* pv. theae, *P*. *amygdali* pv. morsprunorum (99%)
ISPsy37	1215	1	2	IS3	Psa5 (98%), *P*. *amygdali* pv. morsprunorum (96%)
ISPsy24	nd	1	0	IS3	
ISPsy38	1100	0	31	IS30	*P*. *avellanae* (99%), *P*. *coronafaciens* (99%)
ISPsy33	1429	27	20	IS4_ssgrIS4	*P*. *avellanae*, *Ps* pv. theae, *Ps* pv. actinidifoliorum (all 99%)
ISPsy19-like	nd	1	1	IS5	
ISPa67-like	nd	1	1	IS5	
ISPsy2	1200	1	1	IS5_ssgr_IS5	*Ps* pv. eriobotryae, *P*. *savastanoi* pv. phaseolicola (99%)
ISPsy34	2041	11	24	IS66	*Ps* pv. avii, *Ps* pv. actinidifoliorum (96%)
ISPsy43 (Tn6211)	4875	1	0	IS66	*P*. *amygdali* pv. morsprunorum (99%), many *P*. *syringae* pathovars (99%)
ISPsy32	1170	60	48	IS630	Psa2 (99%), *P*. *broussonetiae* (99%), *Ps* pv. actinidifoliorum (98%)
ISPsy25	nd	1	1	IS630	
ISPsy32-like	nd	11	11	IS630	
ISPsy200-like-A	459	1	1	IS200-like	*Ps* pv. theae (99%), *P*. *amygdali* pv. morsprunorum (99%)
ISPsy200-like-B	459	1	1	IS200-like	Ps pv. theae, P. avellanae (99%)
TnE622	3734	1	1	Tn3	*P*. *savastonoi* pv. glycinea B076 (99%)
ISPsy39	3939	0	3	Tn3	*P*. *amygdali* pv. morsprunorum (99%)
ISPsy42	5667	3	0	Tn3	*Xanthomonas arboricola* pv. pruni plasmid pXap41 (99%)
MITEPsy3	102	78	0	MITE	*P*. *amygdali* pv. lachrymans (87%)
PSA_retron1	2566	1	0	retron	
PSA_retron2	2700	1	0	retron	
PSA_retron3	2102	0	1	retron	
Psa_I1	1867	15	0	gp II intron	Psa5 (100%), *P*. *avellanae* (99%),
Psa_I2	1873	13	19	gp II intron	*Ps* pv. theae (99%), *P*. *savastanoi* pv. phaseolicola (99%),
Psa_I3	1873	28	10	gp II intron	*P*. *avellanae*, *Ps* pv. theae, *Ps* pv. actinidifoliorum, Psa5, *Ps* pv. tomato (all 99%)

In general, the most closely similar IS to those found in Psa1 or Psa3 are present within *Pseudomonas syringae* pathovars or the genomes of other plant pathogens such as *P*. *amygdali*, *P*. *savastonoi* or *P*. *avellanae* (Table 2).

The genomes of the Japanese Psa1 and New Zealand Psa3 also contain retron retroelements. Retrons consist of a reverse transcriptase gene and an adjacent inverted repeat sequence corresponding to the overlapping genes for the RNA and DNA segments of an msDNA. They generate multi-copy, extrachromosomal msDNA, which is a molecule of single-stranded DNA joined covalently to RNA^29,30^. The role of retrons remains unclear, some evidence implicates them in virulence of *Vibrio*^31^. Identical or almost identical retrons to the two elements found in the New Zealand Psa3 are found in Chinese isolates of Psa3, where they occupy the same positions in the various Psa3 genomes. This suggests that these retrons are not actively mobile. The Japanese Psa1 genome contains only one retron, which is absent from the Psa3 genome. Toro and Nisa-Martínez^32^ compiled a set of retron sequences from data in GenBank; these included sequences from several *Pseudomonas* species including *P*. *syringae* pv. actinidifoliorum ICMP18883.

Three different types of Group II intron retro-elements are represented in the genome of ICMP18708. Two of these types have almost identical representatives in ICMP9853. These elements are apparently active and are present in different numbers in the two genomes. All three types of Group II intron have similar representatives in other *P*. *syringae* pathovars (Table 2). Group II introns are mobile genetic elements found in many bacterial genomes, including those of several species of *Pseudomonas*^33^ and pathovars of *P*. *syringae*^34^. They consist of a highly structured RNA, which provides the catalytic activity for splicing, and an intron-encoded protein with endonuclease, reverse transcriptase, and RNA maturase activities which provide mobility to the intron^30^.

The large (16,316 bp) transposon, Tn6212, present on the ICEs of many Psa3 and other *P*. *syringae* pathovars^25^ is not found in ICMP9853. Tn6212 occurs at the same site in Pac_ICE1, Pac_ICE2 and PaC_ICE3^25^. It is almost identical to the Tn6212 from *P*. *syringae* pv. syringae B728a (with two SNPs in 16,316 bp). This tyrosine recombinase transposon has apparently integrated at the same target site in numerous different ICEs and plasmids. For example, the complete Tn6212 is present on *P*. *syringae* pv. syringae plasmids pPs7B44 and pPs0170 (accessions KY362373 and KY362372 respectively). The wide distribution of this transposon is probably a further example of horizontal transfer. Firrao *et al*., detected the frequent excision of Tn6212 from Psa3 isolates from Europe^8^. The 4,876 bp transposon Tn6211, also known as ISPsy43, is present on the Pac_ICEs of pandemic Psa3, but not found in the genome of ICMP9853 (Table 2). Both Tn6212 and Tn6211 carry genes likely to encode features related to virulence, such as mercury resistance or methyl-accepting chemotaxis proteins.

## Discussion

Advances in long read sequencing, along with the high accuracy of Illumina read correction, enable very accurate descriptions of the genomes of bacteria such as *Pseudomonas syringae* pv. actinidiae. Here, we have compared the genome of a New Zealand strain (ICMP18708) from the Psa3 pandemic with the complete genome of ICMP9853, a Psa1 strain isolated from Japan in 1984.

ICMP18708 has a chromosome size of 6,555,571 base pairs. This is somewhat different to the previously reported size of another New Zealand isolate, ICMP18884, at 6,580,291 base pairs^33^. The difference is due to an assembly error of ~25,000 base pairs in the original ICMP18884 sequence. There is also a previous size estimate of the ICMP18884 genome based on Illumina and Roche 454 sequencing of 6,523,209^15^. This sequence was a scaffolded assembly containing 69 contigs separated by varying lengths of un-sequenced tracts. In ICMP18708 there is a single plasmid of 74,432 bp. A similar size was reported for this plasmid (74,423 bp) by Templeton *et al*.^34^, and a somewhat different size (~71 kb) was reported by McCann *et al*.^15^. In summary, there is close agreement between the genome assemblies generated with SMRT sequencing.

The Japanese Psa1 isolate, ICMP9853, has a chromosome of 6,439,609 bp, similar to the previous estimate of 6.47 Mb for the ICMP9617 genome^15^. ICMP9853 carries two plasmids of 34,963 bp and 32,947 bp. In the assembly reported by McCann *et al*.^15^, there is a single plasmid of 32 kb. The difference is due to an assembly error in which one of the plasmids has been incorporated into the main chromosome of ICMP9617.

Comparison of the genome organisation in ICMP18708 (Psa3) and ICMP9853 (Psa1) using the progressiveMauve algorithm indicates that there are numerous translocation events distinguishing the two strains. There are a greater number of these events in these Psa isolates than between three isolates of *P*. *syringae* pv. syringae^35^. The translocations in Psa have a distinct pattern; they involve one break point in the left hemisphere of the circular molecule and a break point in the right hemisphere. In addition, the two breakpoints are approximately the same distance from the origin of replication (at the same ‘latitude’). This pattern conserves the polar positions of the origin and terminus of replication. It also ensures that the AIM sequences are correctly orientated. The orientation of the AIM sequences is also conserved irrespective of which *tRNA-Lys* site is used for Pac_ICE integration, because the two *tRNA-Lys* sites lie on opposite DNA strands. Firrao *et al*.^8^, noted a large inversion present in one isolate of Psa3 (CRA.FRU14.08) as compared to another European isolate, CRA.FRU12.29; this inversion involves the whole region between the two *tRNA-Lys* sites. The biological significance of the multiple translocations seen in the present study is not obvious. These multiple rearrangements may be selectively neutral, or it may be that genome structure has an effect on transcriptional regulation^36^.

In addition to genome rearrangements, the New Zealand Psa3 and Japanese Psa1 genomes differ with respect to numerous sequences apparently due to horizontal acquisition from other bacteria. A striking example of this involves the restriction modification systems of the two strains. PacBio/SMRT sequencing permits the detection of global methylation patterns and it reveals that the two strains have different active methylase genes. The New Zealand strain ICMP18708 has two active restriction modification systems, a Type II system recognising CTCGAG sites and a Type I system recognising AGCANNNNNGTC sites. Both of these systems are on sequences of DNA that are completely absent from the Japanese Psa1 strain. In addition, the New Zealand strain has three inactivated restriction systems; a Type III which is inactivated by an in-phase stop and a MITE, a Type II disrupted by two MITEs and an ISPsy28 element, and a Type I system which is inactivated by an ISPsy34. The Type I system is present in an active form in other Chinese Psa3 isolates such as Jilo4 and recognises the sequence GAYCNNNNNCTGC. In ICMP18708, the inactive Type I and Type III R-M systems are located on sequences that are completely missing from the Japanese Psa1 strain. It is likely that these sequences have been acquired by horizontal transmission and subsequently inactivated since other pseudomonads carry very similar restriction modification system genes. The inactive Type II has an inactive homologue in Psa1. There is a full-length putatively functional homologue in Psa6 (biovar6; Genbank accession PBK55636).

The Japanese Psa1 has two active restriction systems; a Type III that recognises the sequence TCCACC, and a Type II system that recognises CTGCAG sites. These genes are located on sequences found uniquely in the Japanese Psa1 genome. In other words, they are probably derived by horizontal acquisition from other bacteria. These restriction systems also have closely similar homologues in other bacteria. In summary, comparison of the two Psa genomes (ICMP9853 and ICMP18708) indicates that there are eight restriction systems that have been acquired by horizontal transfer, four of which have been inactivated. It is plausible that these horizontal acquisitions of restriction systems are selectively advantageous. They allow a bacterium to alter its restriction repertoire and, as a consequence, efficiently escape phage populations. As four of these systems have been inactivated, this may suggest that the presence of too many active methylation systems imposes a selective disadvantage such as in replicative delay. In consequence, when a new restriction methylation system is acquired, the pre-existing one may be selectively eliminated. The presence of these multiple restriction methylation systems does not seem to have prevented the acquisition of Pac_ICEs or plasmids. The Pac_ICEs and plasmids may be able to evade restriction, in part because they are transferred as single stranded molecules. In addition, horizontally transferred mobile elements such as ICE or plasmids may avoid restriction through the presence of anti-restriction proteins^37,38^. In numerous cases of the acquisition of the R-M systems, it is possible to determine a potential probable source of the horizontal transfer. In all the cases found in Psa3 or Psa1, the donor bacterium is from a related plant pathogenic pseudomonad, such as *Pseudomonas syringae* pv. theae or pathovars of *P*. *amygdali*.

The New Zealand Psa3 strain carries an integrative, conjugative element (Pac_ICE1) integrated into a *tRNA-Lys*. European pandemic strains of Psa3 carry a related but distinct ICE (Pac_ICE2) at the alternative *tRNA-Lys* integration site^25^. The Japanese strain does not carry an ICE at either site. In addition to the Pac_ICE, the Psa3 strains carry ICE-derived sequences at both of the *tRNA-Lys* sites (exapt_ICEs). The Japanese Psa1 genome does not carry any ICE-derived sequences at the *tRNA-Lys* sites and comparison of the New Zealand Psa3 genome with the Japanese Psa1 genome permitted the delineation of these ICE derived sequences. These exapt_ICEs are present in all pandemic Psa3. They carry genes likely to be involved in forming a transmembrane pore and other activities associated with a Type IV secretion system (T4SS). The exapt_ICEs are clearly not self-transmissible, since they lack any pilus genes; nor can they be IMEs (integrative mobilisable elements, a class of defective ICEs) since they are not flanked by two *tRNA-Lys* sites and therefore cannot be excised from the genome. The strong conservation of the sequence and order of the exapt_ICE T4SS genes, despite the loss of two thirds of the ICE precursor, argues that exapt_ICEs must be performing some selectively valuable function involving a transmembrane pore. One possibility is that they are involved in the extracellular secretion of DNA (eDNA;^39^), analogous to the systems found in *Helicobacter pylori*^40^ and *Neisseria gonorrhoeae*^41^ or in the secretion of effector proteins^27^.

In addition to the patterns described above, another source of evolutionary change in the two Psa genomes is a consequence of the movement of mobile elements within the genomes. Both genomes carry a wide range of mobile elements; some present at high copy number. Repetitious, mobile elements have been implicated in evolutionary diversification in bacteria^30,42,43^. The movement of these elements may alter the expression level of genes or inactivate them completely (as in the case of the two restriction systems inactivated in ICMP18708), or they may be selectively neutral. Modification of virulence as a result of gene inactivation via IS element insertion have been detected twice among Psa3 recently isolated in Europe^8^. Two Psa3 strains which did not elicit hypersensitive responses and showed limited growth in kiwifruit leaves, were shown to have an insertion of ISPsy31 or ISPsy36 in the *hrpS* or *hrpR* gene, respectively^8^. It is unclear whether these mutations are selectively neutral or confer some selective advantage.

The creation of SMRT/PacBio-based Psa3 and Psa1 genome assemblies allows an accurate description of the repetitious sequences in each genome. These mobile elements can provide a very sensitive method of detecting recombination within the *P*. *syringae* pv. actinidiae pathovar (that is, between biovars such as Psa3 and Psa1). They can potentially transfer from one biovar to another on plasmids or ICEs and, once transferred they would be able to integrate into the recipient genome without requiring homologous recombination. Given these characteristics, they can be viewed as sentinels of horizontal transfer and recombination. In the genome of ICMP18708 (Psa3) there are a total of 162 sentinel mobile elements that are absent in ICMP9853 (Psa1). These comprise the mobile element ISPsy17 found 11 times, the MITE present in 78 copies, the group II intron Psa_I2 (13 copies). The Psa_I3 (28 copies) in the New Zealand Psa3 genome all carry a distinct SNP compared to those in the ICMP9853 genome. Of the ISPsy32 elements, the Psa3 New Zealand genome carries a distinct subgroup, with 32 copies, which is absent from the Psa1 genome. Given that these 162 elements are distributed essentially at random throughout the genome, this indicates that there is a mobile element, capable of acting as a sentinel of recombination, every 40 kb on average.

The MITE is the clearest illustration of this concept of recombination sentinels. The New Zealand strain has 78 copies of the MITE which is also present in Psa3 from China. For example, the ccA strain Hym1 has ~100 copies and ccB strain Jilo4 has ~100 copies. No other Psa carry numerous copies of this MITE. Psa1 strains carry no copies while Psa2 strains carry one or two copies of a different MITE. *Pseudomonas syringae* pv. theae carries no copies. In summary, this MITE is abundant in Chinese Psa3 genomes and is absent from the Japanese Psa1 genome. This suggests that there has been no significant contribution to the Japanese Psa1 genome from any Psa3 strain by recombination. Similar reasoning applies to the mobile elements restricted to ICMP18708. A similar, reciprocal argument applies to the 31 copies of ISPsy38 in the Japanese genome; there are no copies of this element in the Chinese Psa3 genomes. In summary, the mobile elements serve as very sensitive sentinels capable of detecting the presence or absence of recombination between these two biovars because of their abundance, strain specificity and the ease with which they can integrate into a recipient genome. However, the distribution of these elements indicates that recombination has not occurred between members of these two lineages over an extended period of evolutionary time.

It has been suggested^15^, that the comparison of the New Zealand (Psa3) with the Japanese Psa1 and Korean Psa2 genomes reveals a “signature of within-pathovar recombination” (that is, recombination between Psa3, Psa2 and Psa1). The comparison by McCann *et al*.^15^, used assemblies of Illumina and 454 sequence data from the Psa1, Psa2 and Psa3 genomes. The apparent recombination is due, in fact, to errors in each of these three genome assemblies. The co-ordinates of the data displayed in McCann *et al*.^15^, (Fig. 3) correspond to ~1,086,000 to 1,126,000 of the fully assembled Psa3 genome (accession CP012179). The correct assembly of this sequence contains the following mobile elements (in order) two MITEs, an ISPsy32, an ISPsy34, a Group II intron, another ISPsy32, two further MITEs, an ISPsy28, an ISPsy41 and an ISPsy31. This abundance of mobile elements makes any attempt at phylogenetic analyses misguided. If this 40 kb sequence is aligned against our complete Japanese Psa1 genome, it is apparent that there are major structural differences. For example, there is a translocation in the Psa1 genome such that the 40 kb is no longer contiguous; part is at 1,292,000–1,295,000 while the other end lies in the region of 5,497,000 to 5,485,000. About 10 kb of the Psa3 genome in this region, which includes multiple repetitious elements, is not present in the Psa1 genome (although some copies of related elements do occur elsewhere in the Psa1 genome). This is the region that McCann *et al*.^15^, claim contains abundant SNPs, indicating a signature of recombination. In summary, the detection of recombination requires accurate genome sequencing and assembly and is confounded substantially by including regions containing abundant repetitious mobile sequences.

The accurate description of the genomes of Psa3 and Psa1, including their methylation patterns, provides a sound platform for analysing their evolutionary origin and the more recent changes that have developed during the pandemic. In particular, it permits a better understanding of the virulence of both biovars and the emergence of enhanced virulence. The description of the repetitious, mobile elements is a pre-requisite for the correct assembly of the genome, but in addition, these elements are apparently major drivers of evolution. The genes present on the plasmids, ICE and exapt_ICE carried by Psa3 suggest these elements are also implicated in the enhanced virulence of this biovar.

## Methods

### Bacterial strains, culture and DNA preparation

The bacterial strains used in this study were obtained from the International Collection of Microorganisms and Plants (http://www.landcareresearch.co.nz/resources/collections/icmp) or from Dr Giorgio Balestra at Tuscia University, Viterbo, Italy. KW1/ICMP9853 originates from Japan and was isolated in 1984 from *Actinidia deliciosa*^9^. ICMP18708 originates from New Zealand and was isolated in late 2010 from *Actinidia chinensis*^10^. Both Hym1 and Jilo4 are Psa3 isolates from China^18^. *Pseudomonas syringae* pv. theae ICMP3923 (also known as CFBP2353) was isolated by M. Goto, from *Camellia sinensis* (tea) in Japan in 1970. Strains were grown on King’s B agar plates at 26 °C, with long-term storage in 20% glycerol at −80 °C. DNA was prepared from bacteria grown for 72 hours, using the Mo Bio™ Microbial DNA Isolation Kit (GeneWorks NZ, Ltd) as per the manufacturer’s instructions.

### Genome sequencing, assembly and annotation

Pacific Biosciences SMRT (Single Molecule Real Time) Sequencing of ICMP18708 and ICMP9853 was performed by Macrogen (Seoul), using the RS II platform and the P6-C4 chemistry with one SMRT cell per isolate. For ICMP18708, this generated 105,021filtered subreads, with a mean subread length of 9,567; while for ICMP9853 110,541 filtered subreads were generated, with a mean subread length of 8,571. Illumina MiSeq sequencing of both isolates was performed by NZGL (New Zealand). Paired-end sequencing was performed to 250 base pairs. Primary reads were analysed in FastQC^44^, with low quality reads removed and adaptors trimmed using cleanadaptors^45^. Illumina reads were then paired, before being merged with FLASH^46^, giving effective read lengths of 200–500 base pairs.

The initial assembly of Psa ICMP9853 was generated with HGAP 3.0. This assembly was then improved with read mapping of the primary SMRT reads in Geneious^47^, identifying areas that may have been misassembled. These areas were then validated with Sanger sequencing of PCR products; Sanger sequencing was performed by GAS at Otago University (http://gas.otago.ac.nz) using ABI BigDye Terminator version 3.1. Further read mapping was used to generate spanning coverage across such areas. Illumina reads that had been processed and merged as above were used as a final correction for small errors, producing a completed assembly. Automated annotation of the complete genome was then performed using the NCBI Prokaryotic Genome Annotation Pipeline (PGAP).

### Methylome and analysis of restriction systems

The methylome of Psa1 ICMP9853 was determined using the ‘Base Modifications and Motifs’ feature in SMRT Analysis version 2.3.0. In short, the real-time nature of the Pacific Biosciences SMRT Sequences show base modification as a change in interpulse duration (IPD), the time elapsed between incorporation of adjacent nucleotides by DNA polymerase. A comparison is then made to an *in silico* control, enabling a quality value (QV) to be generated for each site potentially carrying a base modification. Details can be found by accessing Pacific Biosciences/kineticsTools on github, the Web-based Git repository hosting service.

The presence of genes encoding restriction enzymes in ICMP18708, ICMP9853 and other isolates was predicted through comparison to datasets on Rebase^48^.

### Comparative genomics

The completed genome of Psa ICMP9853 (GenBank accession CP018202) was compared to the genome of a representative of the pandemic lineage, Psa ICMP18708 from New Zealand (GenBank accession CP012179), using progressiveMauve^49^ as part of the Mauve plugin accessed in Geneious^47^. BLASTn comparison of the completed genome, both in its entirety and specific features such horizontally acquired elements, was also carried out with related *Pseudomonas syringae* genomes via the NCBI website.

The IS and transposon sequences were annotated with the aid of ISsaga (http://issaga.biotoul.fr/ISsaga2). ISsaga is a semi-automatic annotation system directed at insertion sequences (IS)^50^.

The genome sequences of ICMP18708 (Psa3) and ICMP9853 (Psa1) were submitted to the PHAST web server for the identification and annotation of prophage sequences^51,52^. Island Viewer 4^53^ was used as a guide for the identification of genomic islands.

### Accession numbers

The nucleotide sequence data of ICMP18708 are available in the DDBJ/EMBL/GenBank database under accession numbers CP012179 and CP012180, while accession data for ICMP9853 are CP018202, CP018203 and CP018204.

## Electronic supplementary material

Supplementary Figures

## References

[1] Vanneste JL (2013). Identification, virulence and distribution of two biovars of *Pseudomonas syringae* pv. actinidiae in New Zealand. Plant Dis..

[2] Vanneste JL (2017). The scientific, economic, and social Impacts of the New Zealand outbreak of bacterial canker of kiwifruit (*Pseudomonas syringae* pv. actinidiae). Ann. Rev. Phytopathol..

[3] Scortichini M, Marcelletti S, Ferrante P, Petriccione M, Firrao G (2012). *Pseudomonas syringae* pv. actinidiae: a re-emerging, multi-faceted, pandemic pathogen. Mol Plant Pathol..

[4] Chapman JR (2012). Phylogenetic relationships among global populations of *Pseudomonas syringae* pv. actinidiae. Phytopathology.

[5] Marcelletti S, Ferrante P, Petriccione M, Firrao G, Scortichini M (2011). *Pseudomonas syringae* pv. actinidiae draft genomes comparison reveal strain-specific features involved in adaptation and virulence to *Actinidia* species. Plos One.

[6] Mazzaglia A (2012). *Pseudomonas syringae* pv. actinidiae (PSA) isolates from recent bacterial canker of kiwifruit outbreaks belong to the same genetic lineage. PLoS One.

[7] Colombi E (2017). Evolution of copper resistance in the kiwifruit pathogen *Pseudomonas syringae* pv. actinidiae through acquisition of integrative conjugative elements and plasmids. Environ Microbiol..

[8] Firrao G (2018). Genomic structural variations affecting virulence during clonal expansion of *Pseudomonas syringa*e pv. actinidiae Biovar 3 inEurope. Front. Microbiol..

[9] Takikawa Y, Serizawa S, Ichikawa T, Tsuyumu S (1989). Goto, M. *Pseudomonas syringae* pv. actinidiae pv. nov.: the causal bacterium of canker of kiwifruit in Japan. Ann Phytopathol Soc Jpn..

[10] Everett KR (2011). First report of *Pseudomonas syringae* pv. actinidiae causing kiwifruit bacterial canker in New Zealand. Australas. Plant Dis. Notes.

[11] Yee TW, Smith DW (1990). *Pseudomonas* chromosomal replication origins: a bacterial class distinct from *Escherichia coli*-type origins. Proc Natl Acad Sci USA.

[12] Duan J, Jiang W, Cheng Z, Heikkila JJ, Glick BR (2013). The complete genome sequence of the plant growth-promoting bacterium *Pseudomonas* sp. UW4. PLoS ONE.

[13] Bigot S (2005). KOPS: DNA motifs that control *E*. *coli* chromosome segregation by orienting the FtsK translocase. EMBO J..

[14] Hendrickson H, Lawrence JG (2006). Selection for chromosome architecture in bacteria. J. Mol. Evol..

[15] McCann HC (2013). Genomic analysis of the kiwifruit pathogen *Pseudomonas syringae* pv. actinidiae provides insight into the origins of an emergent plant disease. PLoS Pathog..

[16] Fox KL, Srikhanta YN, Jennings MP (2007). Phase variable type III restriction-modification systems of host-adapted bacterial pathogens. Mol. Microbiol..

[17] Seib KL, Jen FE, Scott AL, Tan A, Jennings MP (2017). Phase variation of DNA methyltransferases and the regulation of virulence and immune evasion in the pathogenic Neisseria. Pathog. Dis..

[18] Ciarroni S (2015). Development of a multiple loci variable number of tandem repeats analysis (MLVA) to unravel the intra-pathovar structure of *Pseudomonas syringae* pv. actinidiae populations worldwide. PLoS ONE.

[19] Doberenz S (2017). Identification of a *Pseudomonas aeruginosa* PAO1 DNA methyltransferase, its targets, and physiological roles. mBio.

[20] Chao MC (2015). A cytosine methytransferase modulates the cell envelope stress response in the cholera pathogen. PLoS Genet.

[21] Militello KT, Mandarano AH, Varechrtchouk O, Simon RD (2014). Cytosine DNA methylation influences drug resistance in *Escherichia coli* through increased sugE expression. FEMS Microbiol. Lett..

[22] Balbontín R (2006). DNA Adenine Methylation Regulates Virulence Gene Expression in *Salmonella enterica* Serovar Typhimurium. J. Bacteriol..

[23] Johnson CM, Grossman AD (2015). Integrative and conjugative elements (ICEs): What they do and how they work. Ann. Rev. Genet..

[24] Hastings PJ, Rosenberg SM, Slack A (2004). Antibiotic-induced lateral transfer of antibiotic resistance. Trends Microbiol..

[25] Butler MI (2013). *Pseudomonas syringae* pv. actinidiae from recent outbreaks of kiwifruit bacterial canker belong to different clones that originated in China. PLoS ONE.

[26] Alvarez-Martinez CE, Christie PJ (2009). Biological diversity of prokaryotic type IV secretion systems. Microbiol Mol Biol Rev..

[27] Abby SS (2016). Identification of protein secretion systems in bacterial genomes. Sci Rep..

[28] Maor-Shoshani A, Bacher Reuven N, Tomer G, Livneh Z (2000). Highly mutagenic replication by DNA polymerase V (UmuC) provides a mechanistic basis for SOS untargeted mutagenesis. Proc. Natl. Acad. Sci. USA.

[29] Travisano M, Inouye M (1995). Retrons: retroelements of no known function. Trends Microbiol..

[30] Darmon E, Leach DRF (2014). Bacterial genome instability. Microbiology and Molecular Biology Reviews.

[31] Zimmerly S, Wu L (2014). An unexplored diversity of reverse transcriptases in bacteria. Microbiol. Spectrum.

[32] Toro N, Nisa-Martínez R (2014). Comprehensive phylogenetic analysis of bacterial reverse transcriptases. PLoS One.

[33] Yeo CC, Yiin S, Tan BH, Poh CL (2001). Isolation and characterization of group II introns from *Pseudomonas alcaligenes* and *Pseudomonas putida*. Plasmid.

[34] Templeton MD (2015). Complete DNA Sequence of *Pseudomonas syringae* pv. actinidiae, the causal agent of kiwifruit canker disease. Genome Announc..

[35] Ravindran A, Jalan N, Yuan JS, Wang N, Gross DC (2015). Comparative genomics of *Pseudomonas syringae* pv. syringae strains B301D and HS191 and insights into intrapathovar traits associated with plant pathogenesis. Microbiology Open.

[36] Trussart M (2017). Defined chromosome structure in the genome-reduced bacterium *Mycoplasma pneumoniae*. Nat Commun..

[37] Chen K (2014). ArdA proteins from different mobile genetic elements can bind to the EcoKI Type I DNA methyltransferase of *E*. *coli* K12. Biochim Biophys Acta..

[38] Liang W (2017). Anti-Restriction protein, KlcAHS, promotes dissemination of carbapenem resistance. Front Cell Infect Microbiol..

[39] Ibáñez de Aldecoa AL, Zafra O, González-Pastor JE (2017). Mechanisms and regulation of extracellular DNA release and its biological roles in microbial communities. Front Microbiol..

[40] Smeets LC, Kusters JG (2002). Natural transformation in *Helicobacter pylori*: DNA transport in an unexpected way. Trends Microbiol..

[41] Hamilton HL, Domínguez NM, Schwartz KJ, Hackett KT, Dillard JP (2005). *Neisseria gonorrhoeae* secretes chromosomal DNA via a novel type IV secretion system. Mol Microbiol..

[42] Ooka T (2009). Inference of the impact of insertion sequence (IS) elements on bacterial genome diversification through analysis of small-size structural polymorphisms in *Escherichia coli* O157 genomes. Genome Res..

[43] Stavrinides J, Kirzinger MW, Beasley FC, Guttman DS (2012). E622, a miniature, virulence-associated mobile element. J. Bacteriol..

[44] Andrews, S. C. FastQCv0.11. 3. http://www.bioinformatics.babraham. ac.uk/projects/fastqc/. BabrahamBioinformatics, Cambridge. Published online (2015).

[45] Chatterjee A, Stockwell PA, Rodger EJ, Morison I (2012). M Comparison of alignment software for genome-wide bisulphite sequence data. Nucleic Acids Res..

[46] Magoč T, Salzberg SL (2011). FLASH: fast length adjustment of short reads to improve genome assemblies. Bioinformatics.

[47] Kearse M (2012). Geneious Basic: an integrated and extendable desktop software platform for the organization and analysis of sequence data. Bioinformatics.

[48] Roberts RJ, Vincze T, Posfai J, Macelis D (2015). REBASE-a database for DNA restriction and modification: enzymes, genes and genomes. Nucleic Acids Res..

[49] Darling AC, Mau B, Blattner FR, Perna NT (2004). MAUVE: multiple alignment of conserved genomic sequence with arrangements. Genome Research.

[50] Varani AM, Siguier P, Gourbeyre E, Charneau V, Chandler M (2011). ISsaga is an ensemble of web-based methods for high throughput identification and semi-automatic annotation of insertion sequences in prokaryotic genomes. Genome Biol..

[51] Zhou Y, Liang Y, Lynch KH, Dennis JJ, Wishart DS (2011). PHAST: a fast phage search tool. Nucleic Acids Res..

[52] Arndt D (2016). PHASTER: a better, faster version of the PHAST phage search tool. Nucleic Acids Res..

[53] Bertelli, C., *et al*. IslandViewer 4: expanded prediction of genomic islands for larger-scale datasets. *Nucleic Acids Res*. **10**, 1093/nar/gkx343 (2017). 10.1093/nar/gkx343 PMC5570257 28472413

