# Supplementary material for: Comparison between complete genomes of an isolate of Pseudomonas syringae pv. actinidiae from Japan and a New Zealand isolate of the pandemic lineage
Source: Sci Rep. 2018 Jul 19;8:10915. doi: 10.1038/s41598-018-29261-5 (PMC6053426; doi:10.1038/s41598-018-29261-5)
Supplement: Supplementary file 1 — Supplementary Figures [file 41598_2018_29261_MOESM1_ESM.pdf]

Supplementary Information

Comparison between complete genomes of an isolate of *Pseudomonas syringae* pv. actinidiae from Japan and a New Zealand isolate of the pandemic lineage.

Russell T. M. Poulter, Joycelyn Ho, Thomas Handley, George Taiaroa and Margi I. Butler*
Department of Biochemistry, University of Otago, Dunedin, New Zealand

Supplementary Figures

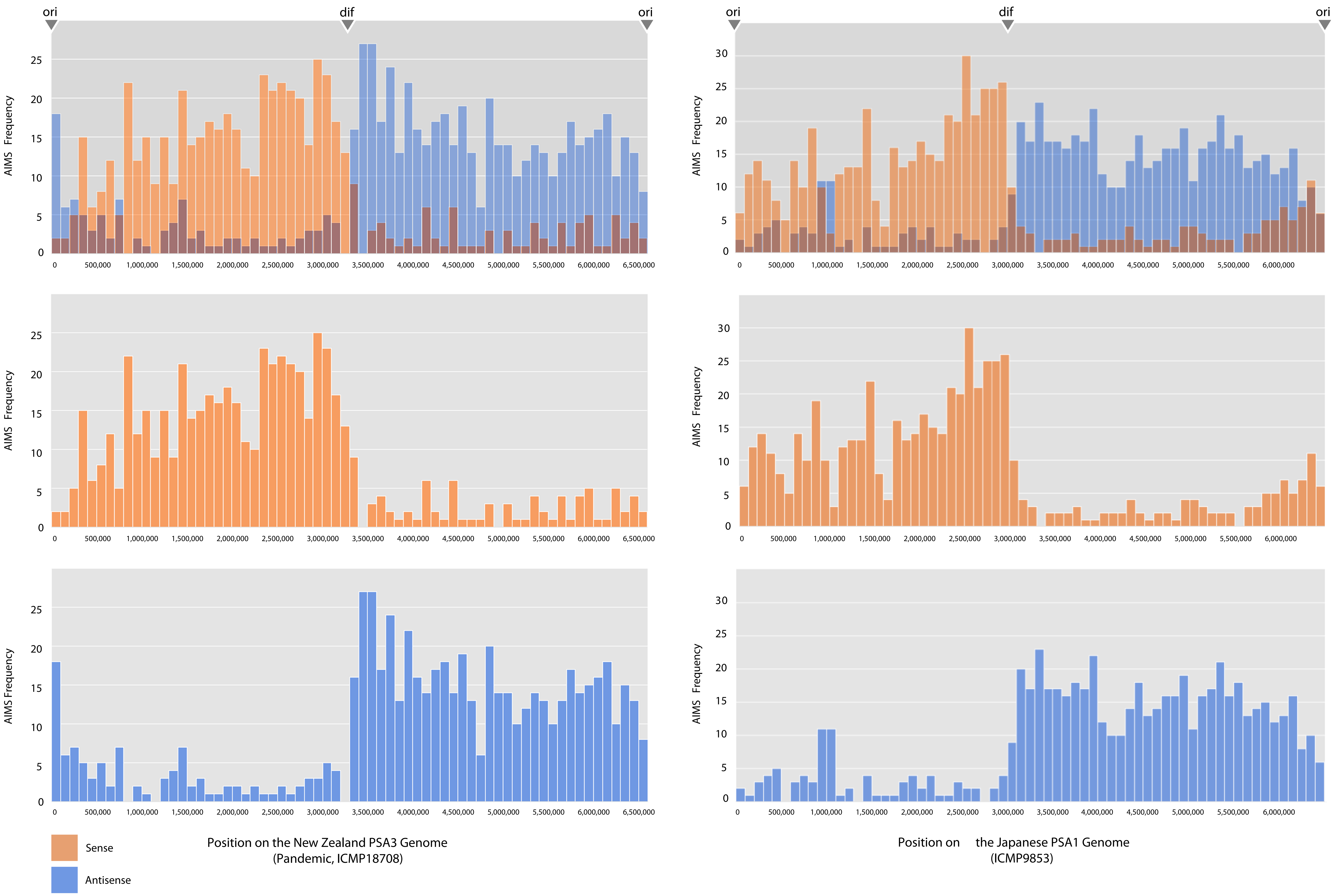

Supplementary Figure 1. The distribution of identified pseudomonad AIMS: left panel New Zealand Psa3, right panel Japan Psa1

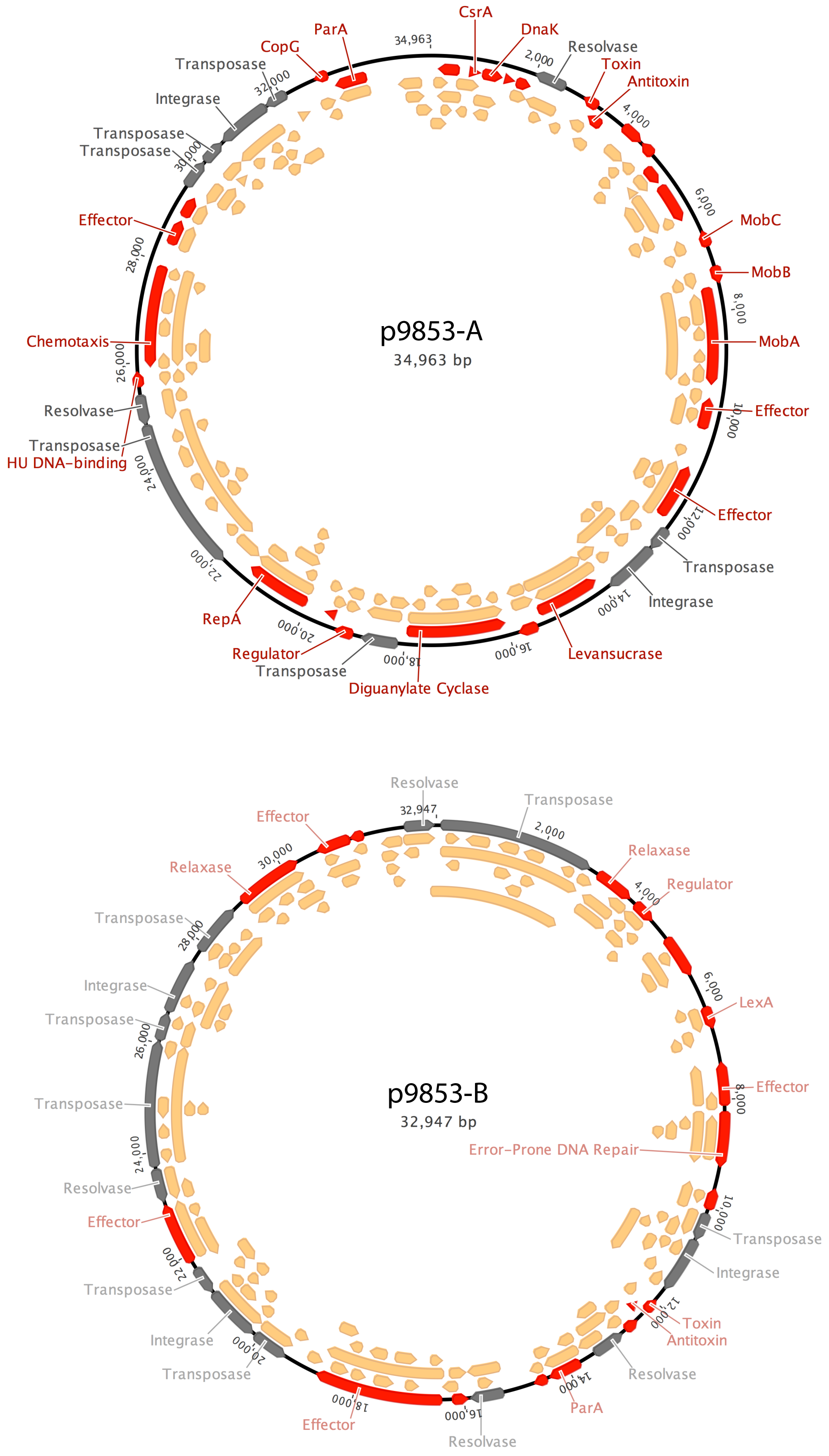

Supplementary Figure 2. The two plasmids from ICMP9853, Psa1.
(Upper panel) p9853-A and (Lower panel) p9853-B. Open reading frames coloured in grey are from mobile elements. In p9853-A there are two ISPsy31, an ISPsy2 and two Tn3-family elements: ISPsy39 and an interrupted TnAs1-like element. In p9853-B there are three ISPsy31, an ISPsy39, a ISPsy2 and other, interrupted IS fragments.

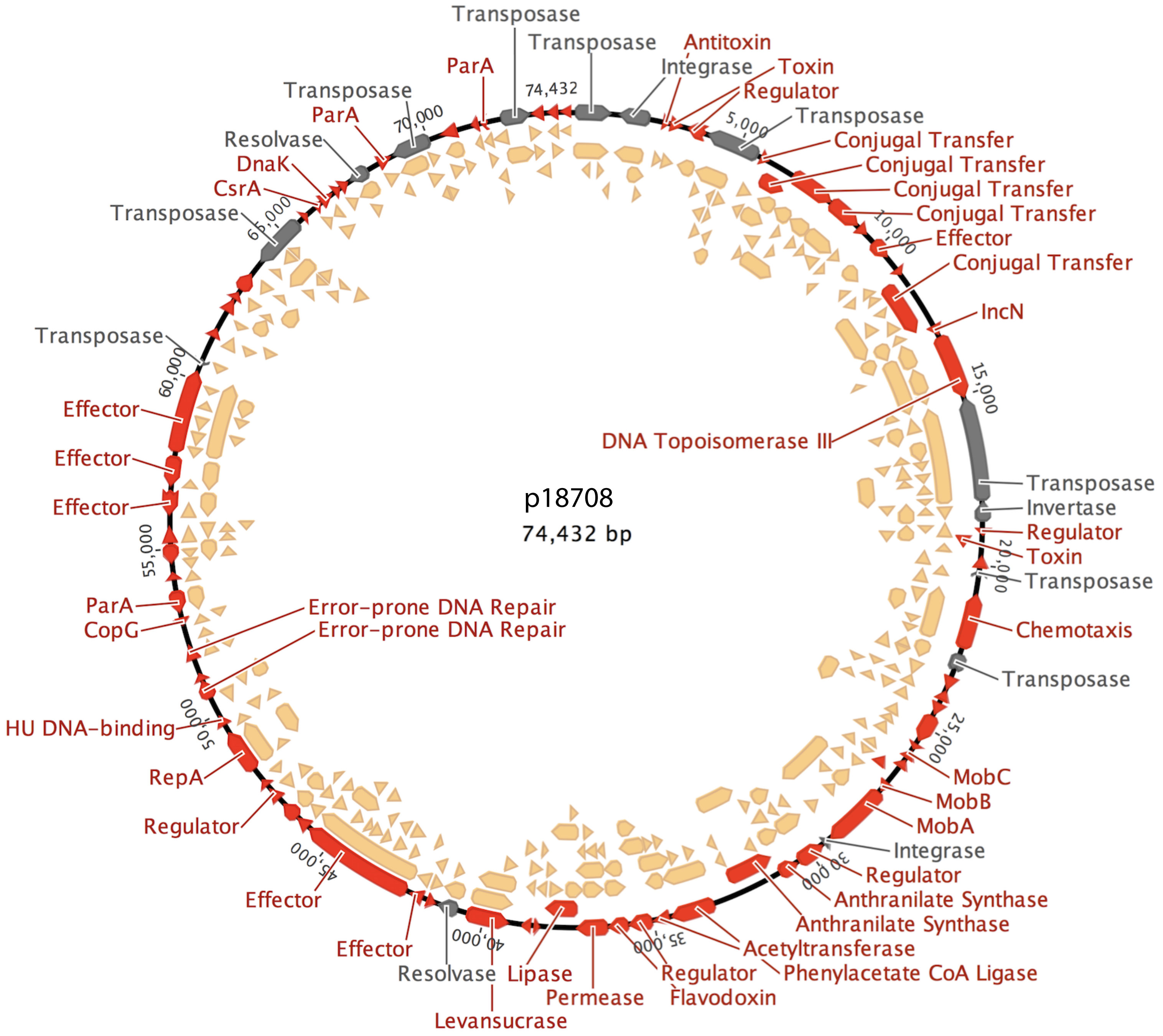

Supplementary Figure 3. The plasmid present in the New Zealand Psa3 genome, p18708; Genbank accession CP012180. Open reading frames coloured in grey are from mobile elements.

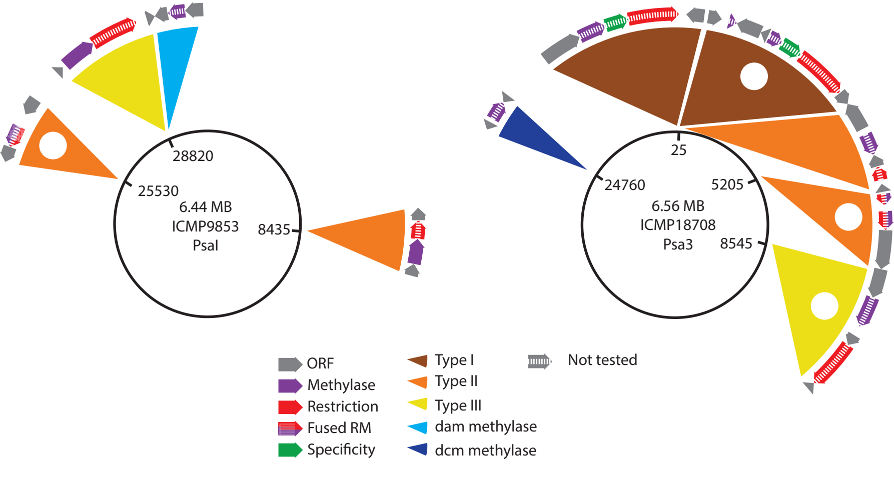

Supplementary Figure 4. The distribution and type of restriction modification systems and DNA methylases in Psa1 (left) and Psa3 (right). Figures adapted from Rebase, http://rebase.neb.com/rebase/rebcit.html

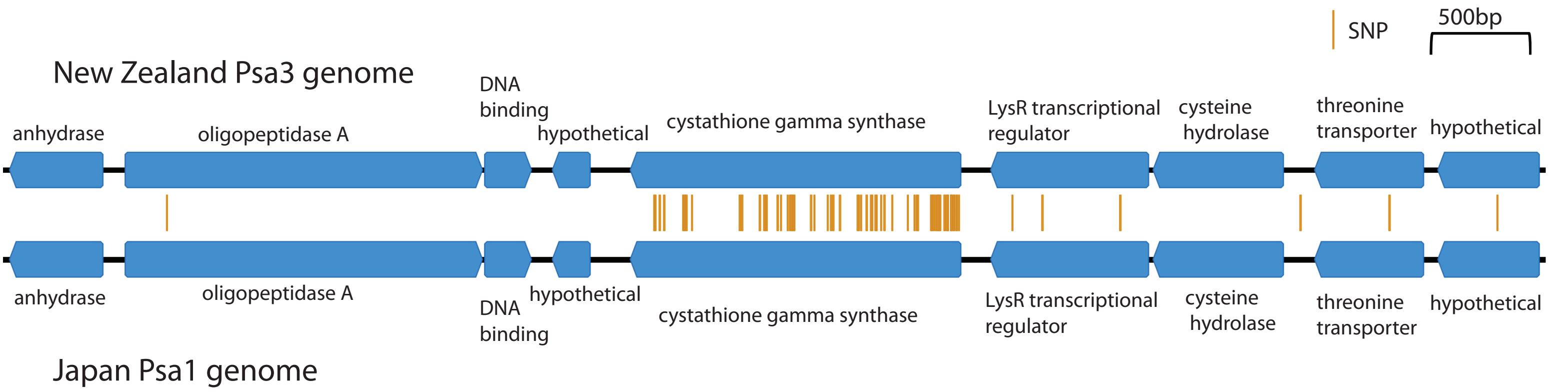

Supplementary Figure 5. Comparison of the sequence at 89877 to 98609 of the ICMP 18708 (Psa3) genome with the homologous region in the ICMP9853 (Psa1) genome. The cystathionine gamma synthase gene (93414-95312) shows 68 SNPs (vertical gold lines) whereas surrounding genes contain few or no SNPs.

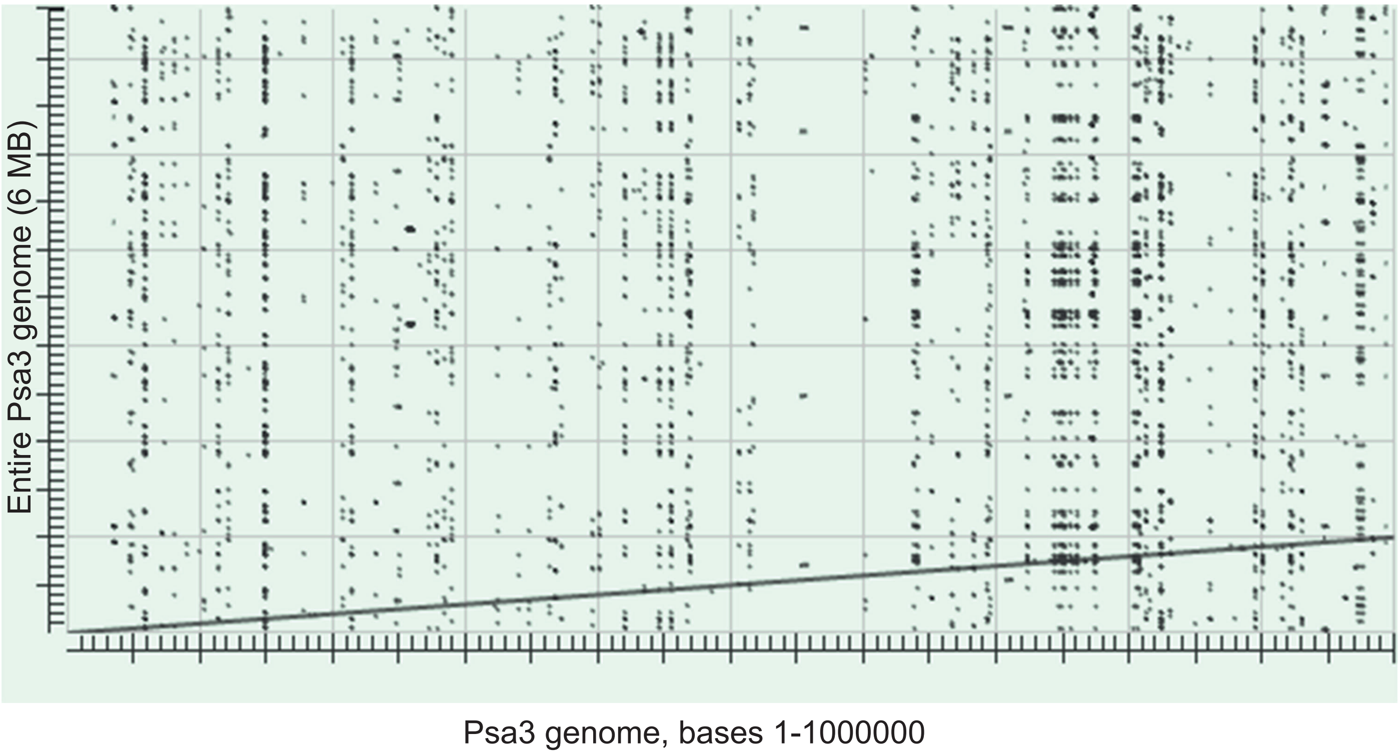

Supplementary Figure 6. Result of a BLASTN alignment of the first 1,000,000 bp of the assembled genome of ICMP18708 (Psa3) (x-axis) plotted against the complete ICMP18708 genome (y-axis), illustrating the abundance of repeated elements in the genome.
